# Forewarning

**Published:** 1887-11

**Authors:** J. W. Fletcher

**Affiliations:** Eastern Star


### FOREWARNING.

It was in '86 while I was lecturing on "General Grant" in Providence, R. I., that I received a most remarkable evidence of spirit presence. My father-in-law, Wm. A. H. Webster, of Lawrence Mass., had been ill for a long time, but no immediate alarm was felt, in fact, he seemed to be gaining if anything, when he was suddenly taken alarmingly worse. On this particular Sunday, I was just going on the stage at Low's Opera House, when I heard a voice say, "The old man dies to-morrow at one o'clock." I started back in surprise for I did not know he was any worse. Again and again it was repeated. My manager tried to laugh me me out of it, and finally I stepped before the people, and in the excitement of the work and the crowd the words were forgotten. As I left the stage, I again heard the same husky whisper repeating the same sad words. Although I was expected to leave for New York, on the midnight train, I determined to go to Lawrence, and wired to the effect that I should be there the next day by one o'clock. My wife received the telegram with some surprise, for she was just writing one to summon me home, as her father had grown so much worse, and was continually calling for me. She went into his room and whispered to him softly, "It is just eight now, Willie wires me saying he will be home at one." The sick man turned very restlessly on his pillow, and then said faintly "I will try and wait. I cannot die without seeing him," and then seemed to drop into a quiet slumber so like the last sleep that more than once they feared he was gone. I arrived home near one o'clock, and hurried into his chamber. They were all kneeling around his bed—the gray haired wife, the young daughter, and others, their faces bathed in tears. At the head of the bed my wife was holding his head upon her shoulder. I placed my hand upon his head where already the dews of death had begun to gather, and asked gently, "Do you know whose hand is upon your head?" He turned his face toward mine with a smile I shall never forget, and murmured, "I shall always know your hand," and then with a sigh fell back upon his pillow dead, and the clock in the adjoining room struck one. "The old man will die at one o'clock," and so he did. Was it not a warning? Let the Seybert Commission explain some of these things that are of almost daily occurrence, and they will render the cause of humanity great service. Let them explain the death bed of Wesley—the vision of Swedenborg, the inspiration of Beethoven, and then we shall perhaps, learn more of the great truth, which now, is but imperfectly understood.

J. W. FLETCHER, *in Eastern Star.*

